# The First Case of Hemoglobin J-Guantanamo Reported During HbA1c Measurement by High-Performance Liquid Chromatography at Moroccan Military Hospital Mohammed V

**DOI:** 10.7759/cureus.50966

**Published:** 2023-12-22

**Authors:** Zohra Ouzzif, Ghita El Moussadeq, Aissam El Maataoui

**Affiliations:** 1 Laboratory of Hematology and Immuno-Hematology, Mohammed V Military Teaching Hospital, Rabat, MAR; 2 Units of Pedagogy and Research in Chemistry, Biochemistry, and Molecular Biology, Faculty of Medicine and Pharmacy, Mohammed V University, Rabat, MAR; 3 Clinical Chemistry, Faculty of Medicine and Pharmacy, Ibn Zohr University, Agadir, MAR

**Keywords:** high-performance liquid chromatography, electrophoresis, hb1ac, hemoglobin j-guantanamo, hemoglobin

## Abstract

We present a 57-year-old woman with diabetes mellitus and no other known comorbidities. HbA1c measurement by high-performance liquid chromatography (HPLC) gave unquantified results and a supernumerary peak was detected on the chromatogram. A thorough exploration of the hemoglobin profile showed the presence of an unclassified variant. Alkaline pH capillary electrophoresis revealed the presence of an abnormal peak migrating at zone 11, comprising 40.1% of total hemoglobin. An abnormal band migrating between hemoglobin A and hemoglobin F was observed by acid gel electrophoresis. Sequencing of the β-globin gene confirmed the presence of a rare hemoglobin variant, hemoglobin J-Guantanamo (HBB:c.386C>A) in the heterozygous state, which was for the first time documented in Morocco. Through this report, we emphasize the importance of careful analysis of the HPLC chromatogram for the detection of possible hemoglobin variants in HbA1c measurement.

## Introduction

Hemoglobinopathies represent one of the most common monogenic diseases worldwide. It has been estimated that approximately 7% of the world's population carries a hemoglobin abnormality, with the highest frequency in sub-Saharan Africa and Asia [1]. Some hemoglobin variants are clinically symptomatic and may be responsible for severe forms. However, about 80% of hemoglobin variants are asymptomatic [2] and are often detected incidentally during glycated hemoglobin (HbA1c) testing in diabetic patients [3]. HbA1c, a product of the irreversible glycation of the beta-globin chain, serves as a biochemical marker for the diagnosis and monitoring of diabetes mellitus. Currently, the most commonly used technique for the measurement of HbA1c is high-performance liquid chromatography (HPLC). This method separates different hemoglobin fractions based on their charge difference and is known to be susceptible to interference from hemoglobin variants [4]. Therefore, several hemoglobin variants have been detected in many cases on the basis of abnormal HbA1c values through the HPLC technique, as other non-chromatographic techniques may not enable their accurate detection [5,6]. In this study, we report the first case of hemoglobin J-Guantanamo that was incidentally detected during HbA1c measurement by HPLC at the Biochemistry-Toxicology Laboratory of Moroccan Military Hospital Mohammed V in Rabat.

## Case presentation

**Figure 1:**
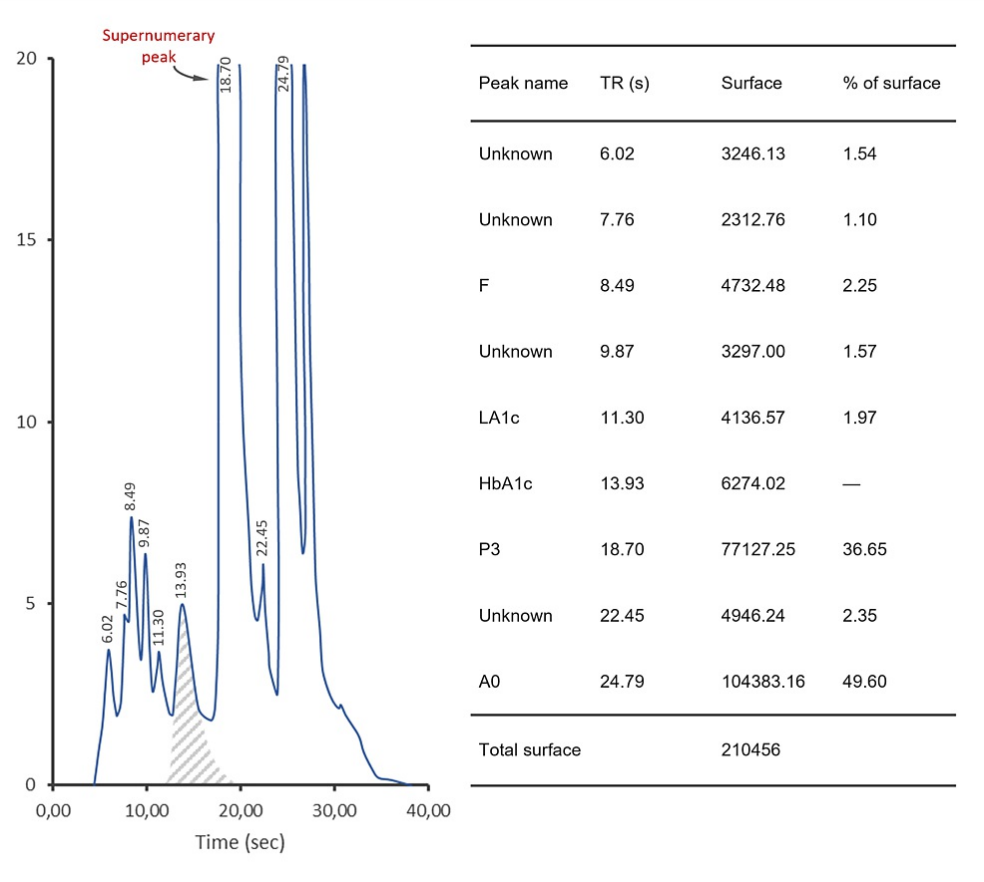
High-performance liquid chromatography (HPLC) analysis of different hemoglobins with an additional peak: chromatographic tracing

**Figure 2:**
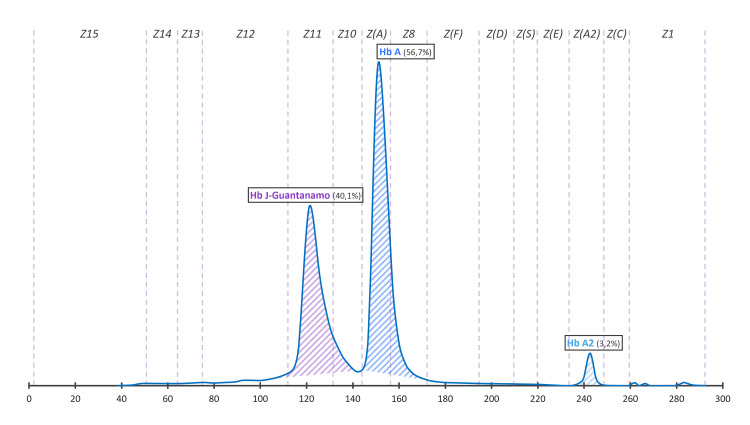
Profiles of electrophoretic examinations of the hemoglobin J-Guantanamo at alkaline pH by the capillary technique using the Capillarys 2 Sebia

**Figure 3:**
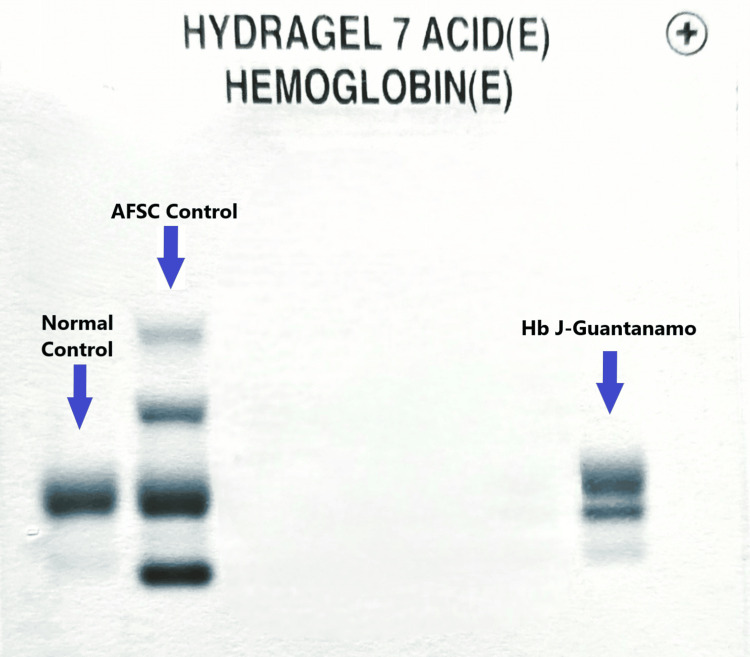
Profiles of electrophoretic examinations of the hemoglobin J-Guantanamo at acid pH on agarose gel using the Hydrasys 2 Sebia

The propositus was a 57-year-old woman from Morocco referred to our hospital for the follow-up of diabetes mellitus. The patient's medical history included a hysterectomy due to metrorrhagia, complicated by an abdominal hernia. The father is diabetic, and there is no consanguinity link with the mother. As part of the follow-up of diabetes mellitus, general analyses were conducted. Fasting blood glucose was measured at 1.34 g/l. The complete blood count showed no specific hematological abnormalities, and the results of the biochemical analyses were within normal ranges (Table 1). However, HbA1c measurement using cation-exchange HPLC on D-100 Bio-Rad yielded unquantified results. Inspection of the HPLC chromatogram revealed a supernumerary peak, raising suspicion of the presence of a hemoglobin variant that could interfere with the HbA1c analysis (Figure 1). In light of these findings, a thorough exploration of the hemoglobin profile was initiated to identify any possible hemoglobin variant. Screening for the hemoglobin variant involved capillary electrophoresis at alkaline pH on the Sebia Capillarys 2 and gel electrophoresis at acidic pH (≃6.2) using the Sebia Hydrasys 2. Alkaline pH hemoglobin electrophoresis on Capillarys revealed the presence of an abnormal peak migrating at zone 11, comprising 40.1% of total hemoglobin (Figure 2). Hemoglobin electrophoresis at acidic pH on Hydrasys showed a migrating band between hemoglobin A and hemoglobin F (Figure 3). These electrophoresis findings indicated the presence of a hemoglobin variant requiring sequencing. Following the patient's written consent, Sanger sequencing of the β-globin gene on Applied 3130XL was performed, confirming the presence of a rare hemoglobin variant, hemoglobin J-Guantanamo (HBB:c.386C>A), in the heterozygous state.

**Table 1:** Laboratory results in the case of hemoglobin J-Guantanamo ALT: Alanine aminotransferase; AST: Aspartate aminotransferase; CRP: C-reactive protein; Hb: Hemoglobin; Ht: Hematocrit; MCH: Mean corpuscular hemoglobin; MCHC: Mean corpuscular hemoglobin concentration; MCV: Mean corpuscular volume; Plt: Platelet; RBC: Red blood cell; WBC: White blood cell.

Parameters	Results	Reference range
Complete blood count		
WBC (10³/μL)	8.1	(4.0 - 10.0)
RBC (10⁶/μL)	4.69	(3.90 - 5.50)
Hb (g/dL)	13.4	(13.0 - 17)
Ht (%)	40.8	(41.0 - 53.0)
MCV (fL)	86.9	(82.0 - 98.0)
MCH (pg)	28.5	(27.0 - 33.0)
MCHC (g/dL)	32.8	(32.0 - 36.0)
Plt (10³/μL)	301	(150 - 450)
Biochemistry		
Sodium (mmol/L)	142	(135 - 145)
Potassium (mmol/L)	4.5	(3.70 - 5.30)
Chlorides (mmol/L)	107	(95 - 110)
Alkaline reserve (mmol/L)	26	(21 - 28)
Glucose (g/L)	1.34	(0.70 - 1.10)
HbA1c (%)	Unquantified	(4 - 6)
Bilirubin total (mg/L)	6	(3 - 12)
AST (UI/L)	20	(< 35)
ALT (UI/L)	18	(< 40)
CRP (mg/L)	7.5	(< 5.0)
Ferritin (ng/mL)	146	(18 - 160)
Haptoglobin (g/L)	1.84	(0.3 - 2)

## Discussion

Hemoglobin J-Guantanamo is an extremely rare and benign variant of hemoglobin. It is formed as a result of a point mutation that affects the 128ᵉ amino acid of the β-globin chain (β-128 [H6] Alanine → Aspartate) at the α1β1 contact area [7]. This zone represents one of the most extensive and important domains in the stability of the hemoglobin tetramer. Hemoglobin J-Guantanamo belongs to the group of unstable hemoglobins. The substitution of alanine (hydrophobic residue) with aspartate (hydrophilic residue) at the α1β1 contact area weakens the stability of the hemoglobin molecule. This promotes the accumulation of free globin subunits, which are themselves unstable, especially α chains. In general, mutations at the α1β1 contact area result in the development of mild hemolytic anemia that is, most often, without apparent clinical consequences [7].

Carriers of hemoglobin J-Guantanamo are rare. This hemoglobin variant was first reported in 1977 in a pregnant woman of black ethnicity from Cuba [8]. Since then, only four other clinical cases have been reported, respectively in China in 1985 [9], Benin in 1988 [10], Chile in 1990 [11], and Japan in 1993 [12]. To the best of our knowledge, our case accounts for the sixth case reported worldwide and the 1st case in Morocco.

In this study, the propositus was heterozygous for hemoglobin J-Guantanamo and showed no particular clinical signs. As for the biological analyses, the patient showed no notable hematological or biochemical abnormalities. The results of our case are generally consistent with other cases of hemoglobin J-Guantanamo reported in the literature. These cases were heterozygous forms, usually asymptomatic and without hematological abnormalities. However, in the case of Cuba and Benin, a slight instability of hemoglobin was observed, related to the nature of the mutation that affects the α1β1 contact area of hemoglobin [8,10]. This instability of hemoglobin J-Guantanamo was manifested by mild hemolytic anemia, mild reticulocytosis, and morphological abnormalities consistent with the presence in the peripheral blood of a high number of target cells [8]. 

In our case, the detection of hemoglobin J-Guantanamo was incidental to the HPLC measurement of HbA1c in the monitoring of diabetes mellitus. The absence of a quantified HbA1c result and the revelation of a supernumerary peak on the chromatogram paved the way for further biological exploration, allowing the identification of the hemoglobin J-Guantanamo variant. Several rare hemoglobin variants were detected in the same manner. In a large study that included 42,371 diabetic patients, 134 patients showed abnormal HbA1c results by the HPLC technique [4]. On the chromatogram, supernumerary peaks were detected and HbA1c values were abnormally low or not quantified. Molecular characterization of these samples allowed the identification of many rare hemoglobin variants in the heterozygous state. Therefore, abnormal or non-quantified HbA1c values and abnormal chromatograms should lead to a thorough exploration of the hemoglobin profile for possible hemoglobin variants.

## Conclusions

In this report, we detected hemoglobin J-Guantanamo incidentally on the basis of unquantified HbA1c results and the presence of a supernumerary peak on HPLC. Although this hemoglobin variant is benign, its report provides important information on the prevalence of the rarest hemoglobinopathies worldwide. Through our case, we emphasize the importance of employing HPLC for the detection of potential hemoglobin variants in HbA1c measurement, particularly the rarer variants. It is crucial for laboratories to be aware of locally occurring Hb variants and to select an appropriate HbA1c testing method. Additionally, in cases where hemoglobin variants are detected, utilizing immunoassay techniques for HbA1c monitoring is advisable, as these methods are not affected by the presence of variants, ensuring consistent and unaffected measurements. Therefore, scrutinizing the HPLC chromatogram for abnormal peaks, coupled with a diagnostic approach aligned with the recommendations of learned societies and expertise, becomes imperative to ensure accurate and reliable results.
